# Benign Episodic Unilateral Mydriasis in a 52-Year-Old Female

**DOI:** 10.7759/cureus.85248

**Published:** 2025-06-02

**Authors:** Prince Darko, Prabina Basnet

**Affiliations:** 1 Internal Medicine, Augusta University/University of Georgia (AU/UGA) Piedmont Athens Regional Medical Center, Athens, USA

**Keywords:** anisicoria, benign pathology, headaches, migraine, mydriasis

## Abstract

Benign episodic unilateral mydriasis (BEUM) is a very rare, under-recognized condition characterized by transient, self-limited episodes of unilateral pupil dilation. It may frequently be part of a migraine process and may present as a diagnostic challenge due to its potential to mimic more serious neurological or ophthalmological conditions. In this case report, we describe the case of a 52-year-old female with a medical history significant for hypertension, anxiety, prior right retinal detachment, and longstanding migraines, who presented with a new onset of recurrent, episodic left-sided mydriasis accompanied by ipsilateral headaches. This paper will outline the findings, medical thought processes, and different diagnoses considered when the patient was assessed in the emergency department and during her hospital stay.

## Introduction

Benign episodic unilateral mydriasis (BEUM) is a rare condition characterized by transient episodes of unilateral pupil dilation, often associated with migraine symptoms. It is so rare that available literature consists primarily of case series and case reports rather than population-based studies. Although considered benign, its episodic nature and neurological manifestations can lead to diagnostic challenges and patient distress. BEUM predominantly affects females and is frequently linked to migraines, with some cases suggesting it may represent a variant of migraine aura [1].

The exact pathophysiology of BEUM remains unclear, but it is hypothesized to involve dysregulation of autonomic innervation to the iris muscles [1,2]. Proposed mechanisms include decreased parasympathetic activity leading to unopposed sympathetic stimulation or migraine-related cortical spreading depression causing autonomic dysfunction [2-5]. Despite these hypotheses, the lack of definitive understanding complicates the ability to predict or prevent episodes.

Clinically, BEUM presents with sudden-onset, self-limiting episodes of unilateral mydriasis lasting from minutes to hours. These episodes may be accompanied by symptoms such as blurred vision, orbital discomfort, headaches, or photosensitivity. Affected patients often exhibit no other neurological abnormalities, and the episodes resolve spontaneously without intervention [3,4,6,7]. Diagnosing BEUM requires a thorough evaluation to exclude other causes of anisocoria, including third cranial nerve palsy, Adie’s tonic pupil, Horner’s syndrome, pharmacological mydriasis, and structural intracranial abnormalities. Neuroimaging, such as magnetic resonance imaging (MRI) and computed tomography (CT), is typically employed to rule out these severe conditions. Once other etiologies are excluded, a diagnosis of BEUM can be confidently made [4-9].

Management primarily involves patient education and reassurance, emphasizing the benign and self-limiting nature of the condition. In cases associated with migraines, addressing the underlying migraine pathology may reduce the frequency or severity of mydriasis episodes. Awareness among clinicians is critical to ensure accurate diagnosis, prevent unnecessary investigations, and alleviate patient anxiety [4,5,8]. As BEUM remains poorly understood, further research is needed to clarify its pathophysiological mechanisms, establish its true prevalence, and develop targeted management strategies.

## Case presentation

A 52-year-old female with a past medical history of migraines, right retinal detachment status post laser treatment, and hypertension presented to the emergency department with complaints of left-sided numbness, headache, and a recurrent dilation of the left pupil. The headache was described as dull and located in the left temporal region, interspersed with sharp pains. She rated the headache severity as six out of 10. It was non-radiating, associated with nausea, and aggravated by movement. She noticed her left pupil was larger than the right when she looked in the mirror after using the washroom. There was no history of slurred speech, difficulty swallowing, facial drooping, limb weakness, loss of consciousness, seizures, or unsteady gait. The patient denied any palpitations, chest pain, fever, ear infection, or recent travel and had not noticed any changes to the size of her pupils in the past.

**Figure 1:**
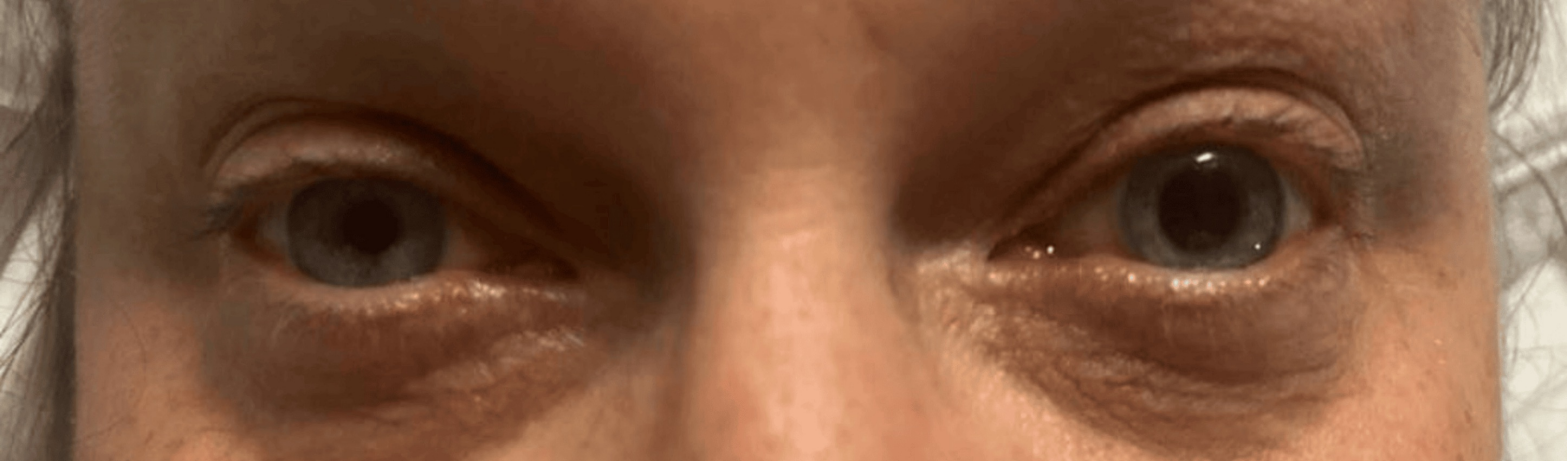
Anisocoria with the left pupil dilated relative to the right pupil, with the picture taken in a well-lit room Written informed consent to include this image in an open-access article was obtained from the patient.

On presentation, the patient's vital signs were stable, with a blood pressure of 120/86 mmHg, a pulse of 60 beats per minute, a respiratory rate of 18 breaths per minute, and an oxygen saturation of 99% on room air. Neurological examination revealed anisocoria, with the right pupil measuring 6 mm and the left 3 mm (Figure 1), both reactive to light and accommodation without evidence of relative afferent pupillary defect (RAPD). The remainder of the cranial nerve examination (II-XII) was normal, with no motor or sensory deficits, and the patient's gait was intact. Cardiovascular and respiratory evaluations were unremarkable.

**Figure 2:**
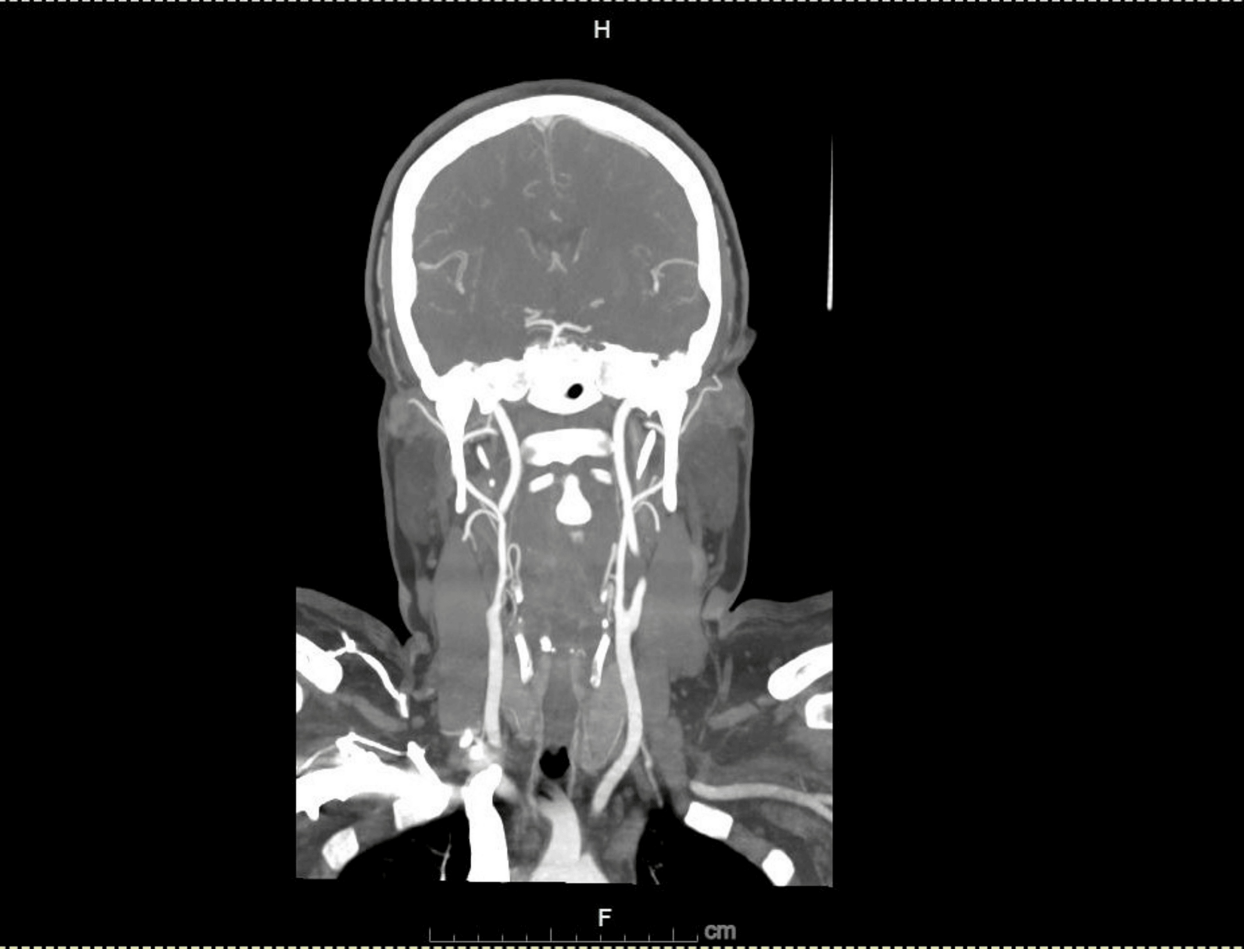
Computed tomography angiography (CTA) coronal view of head and neck showing normal patent arteries

**Figure 3:**
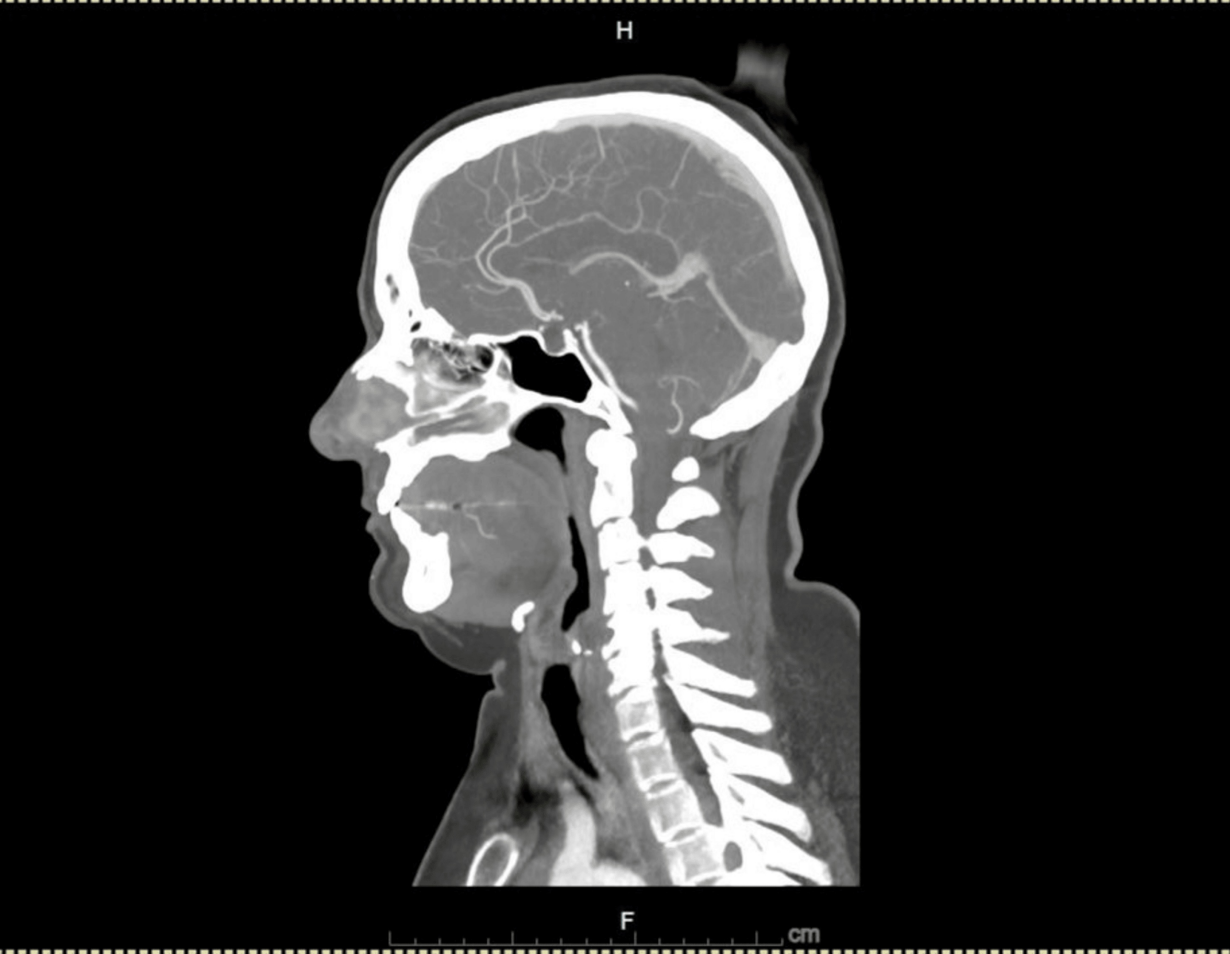
Computed tomography angiography (CTA) sagittal view of head and neck showing normal patent arteries

**Figure 4:**
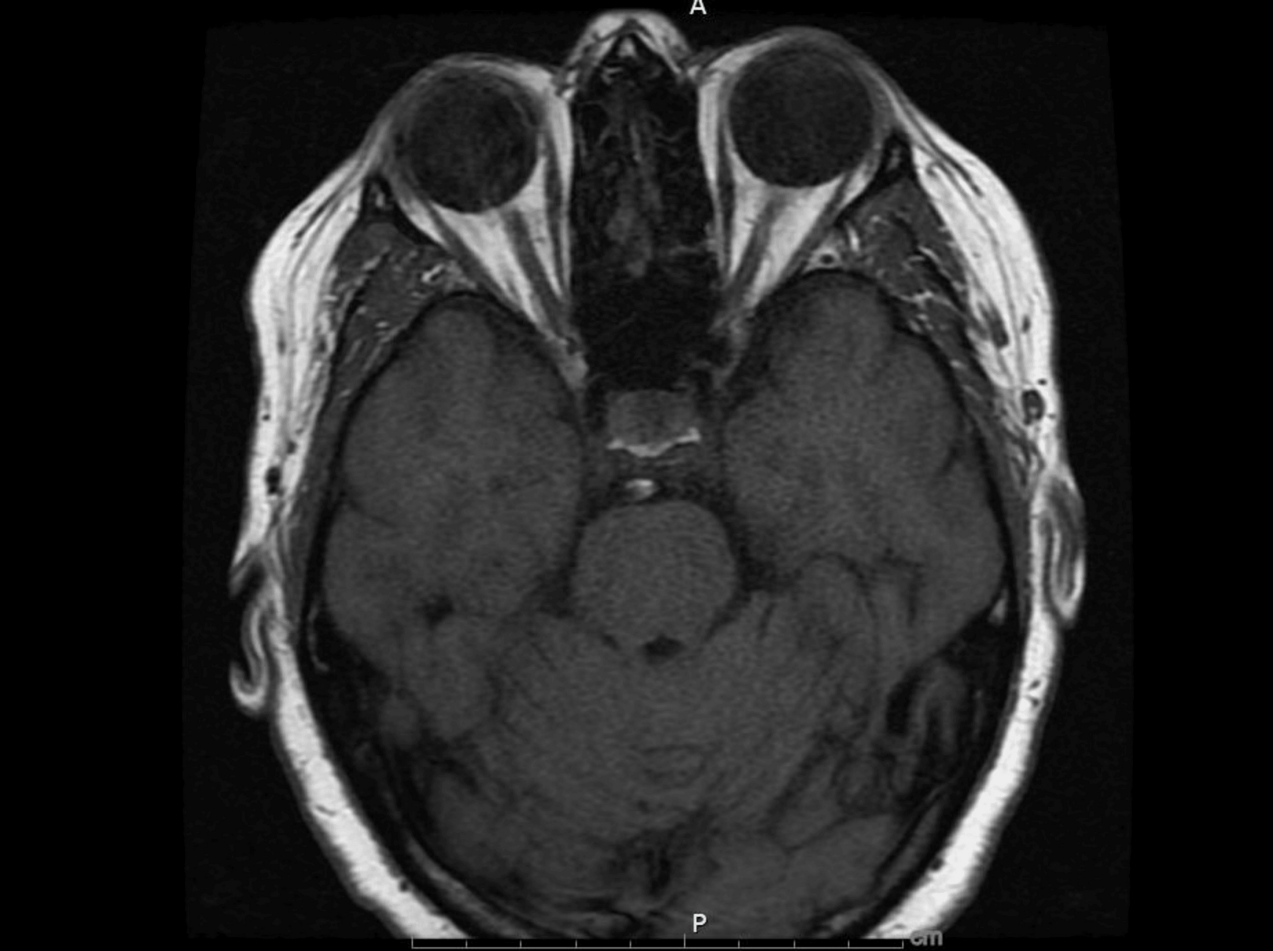
Magnetic resonance imaging (MRI) brain and orbits T1 weighted showing normal anatomy of orbits and retro-orbital structures

**Figure 5:**
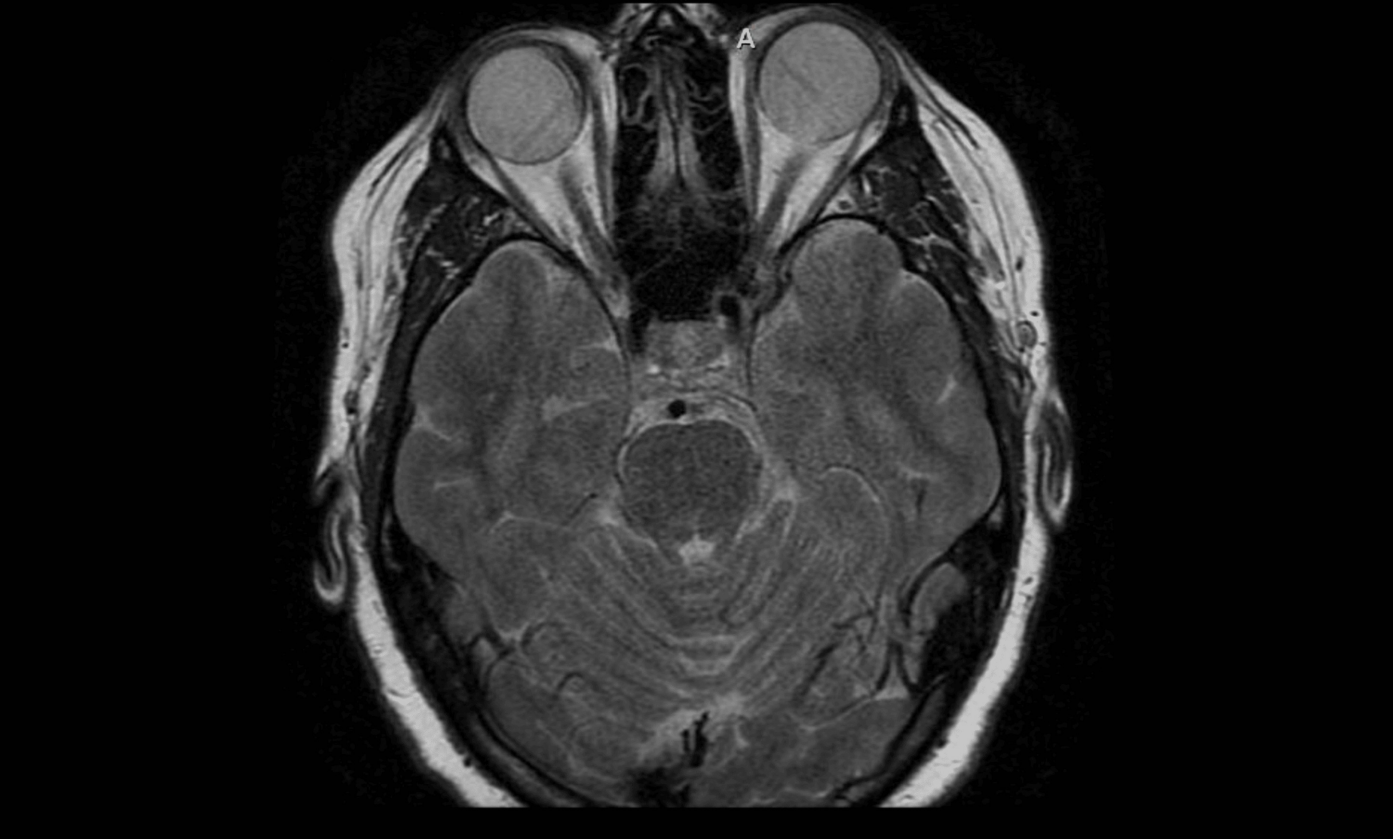
Magnetic resonance imaging (MRI) brain and orbits, T2 weighted, showing normal orbital and brain anatomy

Diagnostic investigations included an electrocardiogram (ECG), which showed a normal sinus rhythm. A CT angiography (CTA) of the head and neck demonstrated patent arteries (Figures 2, 3), with mild beading in the bilateral middle and posterior cerebral arteries, suggestive of atherosclerotic changes. An incidental 1.7 cm hypoattenuating nodule posterior to the left thyroid gland was noted. Laboratory analysis revealed an acute kidney injury (AKI), with a serum creatinine of 1.21 mg/dL and a bicarbonate level of 31 mmol/L (Table 1), likely due to contraction alkalosis in the setting of her nausea and vomiting. MRI of the brain and orbits, performed with and without contrast, showed no abnormalities in the brain parenchyma, ventricular system, or orbital structures (Figures 4, 5).

**Table 1:** Laboratory investigations with results

Parameters	Patient Values	Reference Range
Vitamin B	281	180-914 pg/mL
Sodium	138	133-145 mmol/L
Potassium	3.7	3.3-5.1 mmol/L
Chloride	105	98-108 mmol/L
Bicarbonate	31	22-32 mmol/L
Glucose	130	70-100 mg/dL
Blood Urea and Nitrites	18	7-18 mg/dL
Creatinine	1.21	0.4-1 mg/dL
Calcium	9.6	8.4-10.2 mg/dL
Anion Gap	14	8-16
Thyroid Stimulating Hormone	0.3999	0.5-5.5 mIU/L
Total Cholesterol	168	5-200 mg/dL
Triglycerides	75	0-150 mg/dL
High-Density Lipoprotein	38	Low risk >60, high risk <40 mg/dL
Low-Density Lipoprotein	115	<160 mg/dL
C-reactive Protein	0.55	0.00-1.00 mg/dL
Erythrocyte Sedimentation Rate	9	0-20 mm/HR
International Normalized Ratio	1.01	0.8 – 1.1
Hemoglobin	15.4	12-16 g/dL
Hematocrit	43.1%	36.0-46.0%
Platelets	265	150-350 x 10*3/uL
White Blood Cell Count	6.6	4-11 x 10*3/uL
Glycated Hemoglobin	5.9	4-6%

The differential diagnosis for the patient's presentation of unilateral mydriasis and headache encompassed several conditions, including stroke. The history of migraines and the transient nature of the symptoms aligned with a diagnosis of BEUM. Ophthalmoplegic migraine, characterized by headache with associated cranial neuropathy, was considered but deemed less likely as there were no signs of oculomotor involvement. Intracranial aneurysm or mass lesion was ruled out based on the unremarkable findings from MRI and CTA. Adie’s tonic pupil was excluded, as it typically presents with a pupil that reacts poorly to light, whereas the patient’s pupils were reactive. Carotid artery dissection was considered unlikely given the normal CTA results. Finally, Horner's syndrome was ruled out due to the absence of ptosis and anhidrosis. The patient’s mydriasis improved with transient recurrence, without any active management, and she was discharged on her usual medications for her migraines. These considerations highlight the importance of a comprehensive diagnostic approach in distinguishing between benign and serious causes of anisocoria and headache.

## Discussion

Headache accompanied by anisocoria presents a diagnostic challenge, as it can result from a wide spectrum of causes, ranging from benign to life-threatening conditions [2-4]. This combination of symptoms often requires a thorough investigation to rule out potentially serious neurological or vascular disorders, such as aneurysms, carotid artery pathology, or compressive lesions, which can be associated with significant morbidity and mortality. In the presented case, the patient’s clinical presentation, including her history of migraines, the absence of focal neurological deficits, and unremarkable imaging results, strongly suggested a primary headache disorder rather than a secondary cause.

Migraines, particularly those with autonomic features such as anisocoria, have long been recognized as a potential source of transient pupil dilation [6,7]. As documented by Correia et al., Marfeo A, and Skeik et al., migraine attacks with associated autonomic manifestations, including mydriasis and other signs of sympathetic overactivity, are well-described [1,5,7]. These features are often seen during the acute phase of migraine or as part of a migraine aura, a phenomenon believed to be related to cortical spreading depression (CSD), which may result in altered autonomic regulation and pupil dilation [1,2,5]. In the case of this patient, her prior history of migraine, in conjunction with the transient nature of the anisocoria and the absence of any other neurological deficits, made the diagnosis of a migraine-related phenomenon the most likely explanation for her symptoms. Once more, serious causes, including aneurysms or carotid artery dissections, were excluded through imaging. The diagnosis of BEUM, hence, became a plausible consideration.

The absence of focal neurological signs, such as hemiparesis, dysphasia, or visual field deficits, along with a steady gait, was further reassuring and helped to exclude conditions like ischemic or compressive causes of anisocoria. Valencia et al. emphasized the importance of ruling out such conditions through neuroimaging, particularly MRI and CT, which can identify structural lesions like tumors, strokes, or vascular malformations [2]. In this case, the patient’s unremarkable MRI findings were crucial in ruling out these possibilities, thereby pointing towards a more benign etiology for the patient’s symptoms. The mild beading of the cerebral arteries observed on CTA, attributed to atherosclerotic changes, did not raise concerns for vasculitis or dissection. While these changes were incidental, they reflect common age-related findings in the cerebral vasculature and are generally of no clinical significance unless accompanied by more severe symptoms or imaging abnormalities suggestive of an active pathological process.

The incidental discovery of a thyroid nodule during the patient’s imaging workup warrants further evaluation, but it is unlikely to be directly related to the patient’s neurological symptoms. Thyroid nodules are common and often benign, with many remaining asymptomatic throughout an individual’s life. A comprehensive workup, including thyroid function tests, may be required to assess the nodule's nature and rule out malignancy. Still, there is no evidence to suggest that this finding contributed to the patient’s episode of anisocoria or headaches.

The AKI observed in this patient was attributed to volume contraction, which is a common cause of renal dysfunction in hospitalized patients, particularly those with dehydration, hypotension, or a reduced effective circulating volume. Her AKI resolved with appropriate fluid management and did not require extensive interventions. 

Given the patient’s clinical presentation, the diagnostic findings, and the exclusion of severe conditions and other competing diagnoses, it is likely that her presenting symptoms were due to BEUM, a condition well-documented in the literature. As noted by Woods et al., BEUM is a benign and self-limiting condition typically associated with migraines and autonomic disturbances [6,7]. The transient nature of the anisocoria, combined with the absence of significant neurological findings and the reassuring imaging results, supports this diagnosis. BEUM, although rare, should be considered in the differential diagnosis of any patient presenting with unilateral mydriasis and headache, especially when secondary causes such as aneurysms or vascular pathologies have been ruled out [6-9]. Further studies are needed to better understand the pathophysiology of BEUM and to establish evidence-based management strategies for patients presenting with this constellation of symptoms.

## Conclusions

This case illustrates BEUM as a potential cause of transient unilateral mydriasis in a patient with a migraine history. Awareness of BEUM is important to avoid unnecessary invasive investigations and to provide appropriate reassurance. Given the overlapping features with other neurovascular conditions, a thorough clinical evaluation and targeted imaging are essential to confirm the diagnosis and exclude severe pathology.
